# Vision-Based Module for Herding with a Sheepdog Robot

**DOI:** 10.3390/s22145321

**Published:** 2022-07-16

**Authors:** Virginia Riego del Castillo, Lidia Sánchez-González, Adrián Campazas-Vega, Nicola Strisciuglio

**Affiliations:** 1 Departamento de Ingenierías Mecánica, Informática y Aeroespacial, Universidad de León, 24071 León, Spain; vriec@unileon.es (V.R.d.C.); acamv@unileon.es (A.C.-V.); 2 Faculty of Electrical Engineering, Mathematics and Computer Science, University of Twente, 7522 NB Enschede, The Netherlands; n.strisciuglio@utwente.nl

**Keywords:** computer vision, threat identification, wolf recognition, herding, sheepdog robots, precision livestock farming

## Abstract

Livestock farming is assisted more and more by technological solutions, such as robots. One of the main problems for shepherds is the control and care of livestock in areas difficult to access where grazing animals are attacked by predators such as the Iberian wolf in the northwest of the Iberian Peninsula. In this paper, we propose a system to automatically generate benchmarks of animal images of different species from iNaturalist API, which is coupled with a vision-based module that allows us to automatically detect predators and distinguish them from other animals. We tested multiple existing object detection models to determine the best one in terms of efficiency and speed, as it is conceived for real-time environments. YOLOv5m achieves the best performance as it can process 64 FPS, achieving an mAP (with IoU of 50%) of 99.49% for a dataset where wolves (predator) or dogs (prey) have to be detected and distinguished. This result meets the requirements of pasture-based livestock farms.

## 1. Introduction

Nowadays, people are more concerned about sustainability and biodiversity, and they demand eco-products and healthier food [1]. This trend also helps to enhance regions that have experienced a huge depopulation in recent years. For example, traditional jobs such as sheep herding have become again popular, initiating extensive livestock farming in rural areas as a form of entrepreneurship. Industrial livestock production systems are mainly indoors and have a higher profitability [2]. On the contrary, pasture-based livestock farming increases animal welfare as they can behave naturally and move freely, and it is also positive for biodiversity [3,4]. However, grazing and monitoring the welfare of cattle and sheep is an arduous and time-consuming task, as the animals are often scattered over large areas and require year-round attention [5]. For this reason, assisting the required tasks by deploying autonomous systems is of utmost timeliness, although it presents challenges.

Precision Livestock Farming (PLF) offers technological tools to assist farmers in livestock management [6]. Through the use of sensors and data-driven systems, the herdsmen are able to manage and control several stages of the production flow [7]. In this sense, the benefits of adding new technologies to improve herder productivity are being explored, such as in the case of pasture-based livestock farming. Several different approaches in the literature use certain sensors such as accelerometers, cameras or GPS collars among others to obtain data useful to understand the animal behaviour and monitor them in order to detect diseases, supervise feeding and weight gain control [8].

Pasture-based livestock production remains an important sector in the European Mediterranean basin. It contributes to preserving large agricultural areas of high nature value, which are often located in less industrialised regions with low productive capacity, such as mountainous regions, e.g., in southern Europe [9]. These grazing systems present environmental advantages, facilitating biodiversity and encouraging cultural and landscape diversity [9]. Furthermore the shepherd’s working conditions have personal benefits, such as working in a natural environment. On the downside, they may suffer from inclement weather.

However, pasture-based livestock farming has several trade-offs [9]. Exclusively pasture-feeding animals ensures they maintain nutrient cycles, but it implies landscape modification. Another issue to consider is the prevention of environmental risks, such as wolf attacks. They might indeed harm the herd, causing a reduction in profits. Therefore, the introduction of a mobile robot capable of monitoring the herd, performing grazing tasks and sending information to the farmers when they cannot cover the whole herd would help to improve current PLF solutions. It also helps biodiversity as it can locate wolf packs and send their position to the herder to avoid encountering them.

Robots have applications in many fields as they have a multitude of functionalities such as the ability to swim [10], navigation in dark underground mines [11], assistance in subterranean search-and-rescue [12], and others. In livestock production, many commercial solutions help farmers in their daily routines. One example is robotic milking farms, which allow experts to analyse herd behaviour during the summer, enabling them to control how temperature affects milk production as well as contributing to animal welfare [13]. Other tasks have also been automated to assist farmers, such as feeding robots (https://www.lely.com/solutions/feeding/vector/, accessed on 28 May 2022), forage pushers (https://milkingparlour.co.uk/portfolio/joz-moov-robotic-silage-pusher/, accessed on 28 May 2022), scraping robots (https://www.lely.com/gb/centers/eglish/farm-business-improvement-scheme/, accessed on 28 May 2022) or herd-monitoring robots (https://www.gea.com/en/articles/data-for-better-fresh-cow-management/index.jsp, accessed on 28 May 2022), which provide data to monitor animal welfare, control feeding and prevent disease. However, most of them are used indoors, as they require a specific infrastructure.

Although the use of a four-legged robot as a sheepdog has been reported in the news, it is still an unsolved problem [14]. The reason is basically that the challenges of deploying a robot into the wild (perception of the environment, herd control or communication problems, among others) still requires investigation to allow its use in real-world scenarios. In this work, we focus on the development of a perception system that can build automatically a dataset with the desired predators using the iNaturalist API and determine the presence of such potential threats in order to prevent damages and respecting biodiversity. Otherwise, the proposed system can be deployed on an autonomous robot operating as a sheepdog in an outdoor farm.

We present a vision-based system that provides herders with valuable information from on-site sheepdog robots in real time. This information helps to increase the profitability of the sheep farm by avoiding some threats and helping the shepherd in their daily tasks. The proposed system identifies threats such as the presence of wolves, which helps make decisions about which grazing areas to drive the flock to. Moreover, we have developed a method to automatically build datasets of images of certain potential predators of a region by using the iNaturalist API [15]. The proposed system can be thus adapted to any region, including species of animals specific of a certain area or part of the world.

This paper is organised as follows. In Section 2, the related works are discussed. Section 3 presents the proposed system. The dataset considered to conduct the experiments and all the experimental setup is given in Section 4. Section 5 shows the obtained results. Finally, Section 6 includes a comparison with existing techniques, and Section 7 gathers the achieved conclusions.

## 2. Related Works

PLF involves data-driven systems to control animals, supervising all the related aspects such as their welfare or health, improving the production process. As sensor technology has advanced enormously in recent years, many measures related to physiological, behavioural and productivity aspects can be acquired [16]. Most of them are focused on monitoring and tracking animal behaviour [17,18] as it is related to livestock diseases and also allows for the analysis of extensive and hilly pastures [19].

Different sensors are employed to develop these tasks such as GPS, cameras, accelerometers or thermographic devices [20,21]. Generally, these sensors are classified in two groups: sensors worn by animals such as ear tags, collars, leg straps or implants [5], and sensors placed in the animal’s surroundings as in the case of cameras. Sensors placed on animals have several drawbacks. For example, the use of GPS collars can harm animals or even become stuck in forest. This is solved with sensors placed in the environment, which have more advantages by allowing the tracking of many animals simultaneously instead of a single one.

Among wearable sensors, there are approaches that use accelerometers or gyroscopes. These sensors are attached to the ear or collar to classify grazing and ruminant behaviour in sheep, providing information about their health or even detecting the lack of pasture quality [22].

With non-wearable sensors, in [23], a system for tracking sheep that detects if they are standing or lying with infrared radiation cameras and computer vision techniques is proposed. Using video cameras and deep learning, wild animals can be successfully identified, counted and described [24,25] as well as other particular species such as Holstein Friesian cattle [26]. A quadcopter with a Mask R-CNN architecture has been used to detect and count cattle in both extensive production pastures and in feedlots with an accuracy of 94% [27,28]. A complete review of the use of images from satellites, manned aircraft and unmanned aerial systems to detect wild animals is given in [29].

Regarding herding, most of the existing approaches are based on monitoring animals from a distance, since it results in a performance increase of the livestock farm, improves animal welfare and reduces the environmental impact. This is called Virtual Fencing (VF), and it is used for handling free-ranging livestock, as it optimises the rangelands and acts like a virtual shepherd [30]. By combining GPS information from the collars and remote sensing, it is possible to monitor animal interactions with the landscape to predict animal behaviour and manage rangelands, protecting those regions that are sensitive or likely to suffer degradation due to overgrazing [31]. Other approaches use drones to herd and move animals, especially in areas dangerous for herders [5,32]. Regarding the impact of drones to animals, there are studies that conclude that terrestrial mammals are more tolerant to unmanned aircraft systems [33], becoming accustomed to them [34], but other species, such as penguins, react differently [35]. Moreover, while unmanned ground vehicles (UGVs) can operate in adverse weather conditions, drones cannot and have problems in forested areas [36].

As the proposed system is developed outdoors, wireless coverage is not guaranteed. In [37,38], a review of the existing wireless sensor networks that can be employed in Precision Agriculture is gathered. Among them, the Long-Range Radio (LoRa) Protocol is discussed as well as its use in different approaches such as smart irrigation systems or the monitoring of agricultural production [38,39].

The proposed method goes a step further, focusing not only on the herd but also on potential threats such as the presence of predators such as wolves, which is a major challenge as it involves both livestock safety and wolf conservation [40,41]. In other regions, predators are jaguars [42] or bears [43,44]. Thus, an adaptive method should be automatically configured to work with different species of predators. In Section 4.1, we show how to configure the method to detect other predators. In addition to this, contact between wildlife and livestock can also be studied as it can potentially transmit zoonotic diseases [45].

In order to detect the presence of wolves, computer vision techniques are employed. Traditionally, features such as Scale-Invariant Feature Transform (SIFT), Speeded Up Robust Features (SURF) or Histogram of Oriented Gradients (HOG) were extracted from images and then were classified using Support Vector Machines, among other classifiers, in order to detect objects in an image [46]. These HOG features have been employed, i.e., for human detection [47], animal detection to avoid animal–vehicle collisions [48], and also in real time [49].

Modern approaches use deep learning techniques such as Convolutional Neural Networks (CNN) that extract features from images, which were subsequently classified using several dense layers. There are also solutions that combine traditional features such as HoG with CNN [50,51]. An extensive review of the object detection techniques using deep learning is provided in [52]. Different CNN architectures are used for object detection and classification, which are trained for large datasets—a time-consuming task [53]. As it is not possible to handle certain problems if the available dataset is not so large or the hardware requirements do not allow obtaining results in a short period of time, a technique named transfer learning is used to take advantage of those existing models that have been trained to detect certain objects and adapt them to a different domain [54].

Through transfer learning, pre-trained models can be adapted to new domains in a way that takes advantage of the knowledge extracted from the set of millions of images on which the network was trained and adapts it to the new problem [54]. They also reduce training time as only the added layers, which introduce the information of the particular problem, are trained. The model layers are frozen or fine-tuned, so the existing knowledge is maintained but adapted to the new problem. Most of the popular solutions for object detection are: YOLO (You Only Look Once), Single Shot Detector (SSD) and R-CNN. The existing architectures with the pre-trained models have been applied to animal classification with an accuracy over 90%. Some examples are fish classification [55], bird classification [56], camera trap images classification [57] or wild animal image classification [39].

## 3. System Architecture

The imitation of sheepdog behaviours with a shepherd robot requires a decision-making system that is able to manage information collected by its sensors and to generate actions through its actuators, taking into account the changing characteristics of the environment. As this system will be deployed in a real environment, some conditions, such as lighting and obstacles that must be detected for a proper navigation, are not fixed, since they vary over time. Moreover, these decisions have to offer long and short-term opportunities to be reactive to any expected and unexpected behaviour in the scenario.

This research uses MERLIN [58], which combines deliberative and behavioural capacities. Deliberative capacities define the characteristics of planning to infer long-term tasks. Behavioural capabilities provide the set of actions capable of responding to changes in the environment more quickly. Thus, Merlin allows us to set a certain goal but is also able to react if an unexpected event occurs.

Once the robot is deployed in a real context, it is crucial to handle unexpected behaviours, and for this, the use of different sensors is required. Traditionally, the literature proposes the use of camera sensors, such as the one proposed in this research. This sensor provides information about the context, such as dangerous species, identification of certain animals or predator attacks. The information provided by the proposed method in the robot’s closed environment will promote alternative navigation routes, keep herd control updated or trigger an alarm process to alert the herder remotely if a wolf is detected. The proposed vision module can also be deployed in fixed cameras near grazing areas.

**Figure 1:**
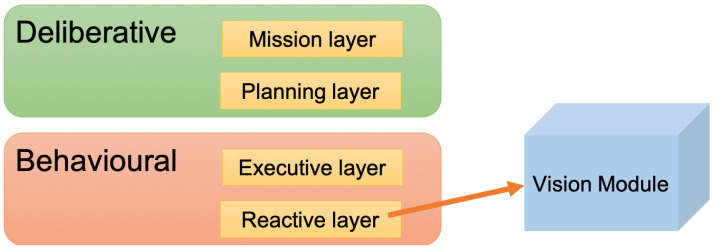
MERLIN architecture and proposed vision module.

The proposed vision module is included along with the other reactive components. It assumes a background approach, feeding a knowledge database and keeping the deliberative monitoring system that would interrupt the active task up to date. The architecture proposed in [58] requires to be updated by including the new vision-based module proposed in this paper in the reactive layer. As this reactive layer gathers all the sensors of the robot, with the proposed vision module, the images acquired by the robot camera are processed. The obtained information is sent to the rest of the layers of the architecture. The useful provided knowledge about what the robot has seen helps the rest of the architecture to make decisions about the subsequent actions to carry out (see Figure 1).

In some scenarios, the autonomous features of the robot are not sufficient for the continuous monitoring of the herd, and there are some specific events where the herder needs to access the robot in real time to monitor dangerous external contexts or improve productivity. The point of view of the sheperd and of the sheepdog are not always the same, so the robot can respond to the commands of the shepherd, who has previous experience and knows how to deal with situations such as weather conditions or grazing specific areas. Thus, images from the robot can be sent to the shepherd’s mobile phone on demand.

The acquired images are processed in the vision module in order to obtain information about the environment. This information is sent to the shepherd by using the LoRaWan® networking protocol. It is widely used in those areas where there are not wireless connections satisfying Internet of Things requirements. The LoRaWan® network architecture allows bi-directional communication, and messages are sent to a gateway that functions as a bridge to the Internet network [59]. Those gateways can be located at different points of the region, making it possible to cover a huge area (≈15 km).

## 4. Vision Module

The vision module belongs to the perception system, which plays an important role in the behaviour of a robot. The use of a Unitree A1, which is a robot dog, has been proposed to detect victim and pedestrians in emergency rescue situations by using thermal and color cameras [60]. These systems usually use a Robot Operating System (ROS) with a vision module to acquire images and detect the existing objects, i.e., smart glasses [61] or cameras on drones [62]. Cameras can also be used to help in robot navigation, exploration and mapping as in [63,64].

**Figure 2:**
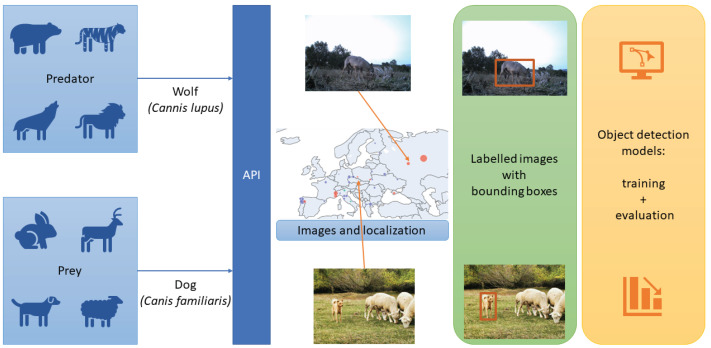
Pipeline of the vision module. First, images and their location are acquired using an API; then, images are labelled to train object detection models.

We propose a vision module that can be used in fixed cameras near pastures and villages or in cameras built into SheepDog robot systems. Figure 2 shows the complete pipeline of the process, where images are acquired and labelled in order to train the object detection models.

There are datasets that are very frequently used in object detection problems [65]. PASCAL Visual Object Classes (VOC) [66] includes 20 categories with animals such as birds, cats, cows, dogs, horses and sheep. ImageNet [67] provides an important source of data for object detection with 200 labelled categories in the 2017 version. Common Objects in Context (COCO) [68] includes the localisation, label and semantic information for each object in one of the 80 categories. In this work, the proposed system can automatically generate species-specific animal datasets through an API.

### 4.1. Data Acquisition and Labelling

Shepherds have to deal with predator attacks on the herd, so it is necessary to anticipate this situation by evading the threat and distinguishing if there is a potential risk [69]. We consider the presence of predatory animals as a potential risk, which can be a bear, tiger, lion or wolf. In the proposed system, prey animals are distinguished from predators to determine suitable grazing areas to maintain distances from predator locations.

We focus on a predatory species of the northwest of the Iberian Peninsula: the Iberian wolf. In [70], a study of the diet of the Iberian wolf shows that it tends to eat goats, cattle, sheep and rabbits, which are some of the animals that farmers raise in the area. Iberian wolves have phylogenetic proximity to other European wolf populations (*Canis lupus*), being considered as a sub-specie of it (*Canis lupus signatus*). Otherwise, dogs are the domesticated descendants of the wolf, presenting similarities as specie (*Canis familiaris*). In this paper, we have created a dataset to differentiate a predator (wolf) from a prey (dog) that can be implemented for more species diversity.

**Figure 3:**
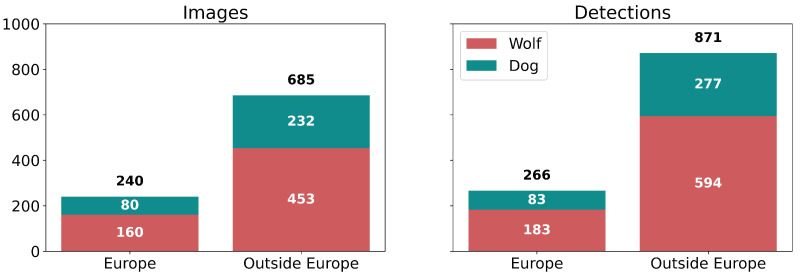
Information of the dataset disaggregated by species and location in number of images (**left side**) and number of detections (**right side**).

We have used the iNaturalist API [15] to create the dataset, obtaining images from two species: *Canis lupus* (wolf) and *Canis familiaris* (dog). As the code is available in [71], the vision module can be adapted to other predators of other regions by using the notebook get_inaturalist and choosing the desired species. Localisation has allowed us to divide the images into two groups: Europe and Outside Europe. Then, images were labelled manually by experts, removing images with low quality. Figure 3 shows how 925 images and 1137 detections are split by species and location.

**Figure 4:**
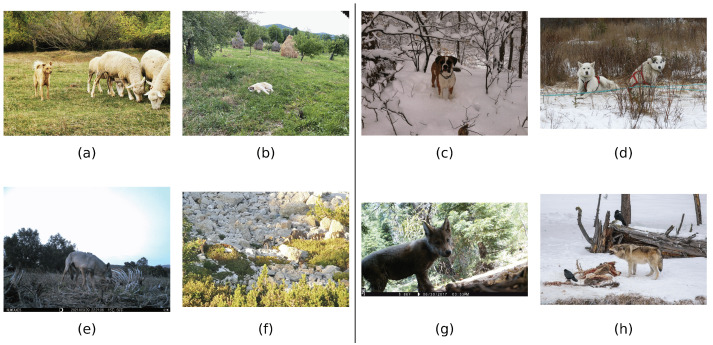
Samples of dogs (**upper row**) and wolves (**bottom row**) in Europe (**left**) and the rest of the world (**right**).

**Figure 5:**
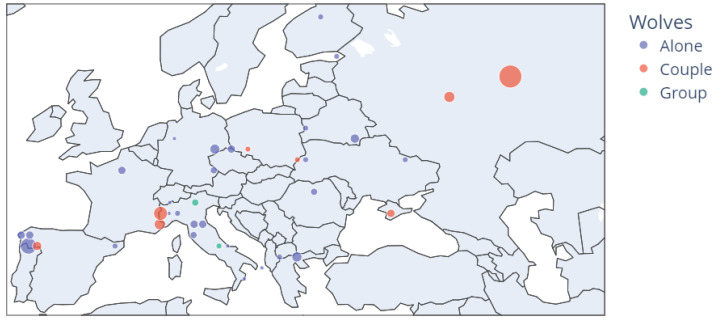
Iberian wolves detected in Europe. Bubble size depends on the number of images.

Images present diversity, as different animals can appear on the images (Figure 4a), lying (Figure 4b), looking to the camera (Figure 4c), partially occluded (Figure 4d), with different lighting conditions (Figure 4e), with multiple detections (Figure 4f) or just one (Figure 4g), feeding (Figure 4h) with different illuminations and different distances from the camera. Due to this information, in Europe, we can observe wolves in couples or alone, as can be shown in Figure 5.

### 4.2. Object Detection Architectures

Object detectors are evaluated based on accuracy, speed and complexity. Two-stage detectors have two steps: extract features from the input image (feature extractor) and recognise the features (classifier). Meanwhile, one-stage detectors combine the feature extractor and classifier into one, reducing complexity and improving speed, but accuracy may be reduced. As the proposed module is deployed in real-time environments, it is based on one-stage detectors. The considered state-of-the-art algorithms [72] based on one stage are You Only Look Once (YOLO) in different versions (YOLOv1, YOLOv2, YOLOv3, YOLOv4, YOLOv5) and Single-Shot MultiBox Detector (SSD). SSD has improved versions such as Deconvolutional SSD (DSSD) that includes large-scale context in object detection, Rainbow SSD (RSSD) that concatenates different feature maps using deconvolution and batch normalisation [73], and Feature-fusion SSD (FSSD) that balances semantic and positional information using bilinear interpolation to resize feature maps to the same size to be subsequently concatenated [74]. The comparison of different architectures for real-time applications presented in [75] also mentions RetinaNet because it has higher accuracy, but it is not recommended for real-time applications, as it has a frame rate lower than 25 frames per second (FPS). EdgeEye [76] proposes an edge computing framework to analyse real-time video with a mean of 55 FPS as inference speed.

For evaluation, different metrics have been considered. First, Intersection over Union (IoU) measures the overlapping area between the predicted bounding box and the ground-truth divided by the union of the areas. IoU is fixed by a threshold (*t*) generating the confusion matrix as:

- True Positives (TP) are those objects detected by the model with an IoU greater than the considered threshold (IoU≥t);
- False Positives (FP) are the detected objects whose IoU is less than the fixed threshold (IoU<t);
- False Negatives (FN) stand for those objects that are not detected;
- True Negatives (TN) are the number of objects detected by the model when actually the image does not have such objects.

Detector models use performance metrics computed from the confusion matrix mentioned above as follows:

- *Precision*: measures how many of the predicted outputs labelled as true predictions are correctly predicted:
  (1) $$Precision=\frac{TP}{TP+FP}$$
- *Recall*: measures how many of the real true predictions are correctly predicted:
  (2) $$Recall=\frac{TP}{TP+FN}$$

In order to compare the results of different authors, there are well-established metrics based on mean Average Precision (mAP), which is the average of the accuracy obtained in the object detection over all the dataset categories. Specifically, metrics are related to datasets mentioned previously [77]:

- COCO metric ($mAP_{COCO}$ or mAP@50:5:95): evaluates 10 IoUs between 50% and 95% with steps of 5% of mean Average Precision as
  (3) $$mAP_{COCO}=\frac{mAP_{0.50}+mAP_{0.55}+mAP_{0.60}+\cdots +mAP_{0.95}}{10}$$
- PASCAL VOC metric ($mAP_{VOC}$ or $mAP_{50}$): evaluates IoU at 50%.

### 4.3. SSD

SSD is composed by two components: a backbone model (in this case, a pre-trained VGG16) and an SSD head with convolutional layers to obtain the bounding boxes and categories of the detected objects. From an image of 300 by 300 pixels, SSD achieves 72.1% $mAP_{VOC}$ on a VOC2007 test at 58 FPS on a Nvidia Titan X [78]. The model has been trained from the PAZ (Perception for Autonomous Systems) library with the object detection module [79].

### 4.4. YOLO

YOLO is also based on a classification backbone with new headers to obtain the bounding box and the assigned class of the object. There are multiple implementations of the architecture, for example, YOLOv3 [80], which uses a DarkNet framework and makes predictions in three different scales. It achieves 57.9% $mAP_{VOC}$ in 51 ms (around 20 FPS). An improvement was made with YOLOv4 [81] obtaining 65.7% of $mAP_{VOC}$ and a speed of 65 FPS. After this, a tiny version of YOLOv3 [82] was proposed with higher speed but lower precision. The newest version, which is YOLOv5 [83], can achieve 68.9% of $mAP_{VOC}$ with more than 80 FPS. Moreover, it includes different versions of complexity, obtaining more precision and more inference time when the model is more complex. The YOLOv5 models are: Nano (YOLOv5n), Small (YOLOv5s), Medium (YOLOv5m), Large (YOLOv5l) and Extra-Large (YOLOv5x).

## 5. Results

The goal of the following experiment is to establish which of the existing architectures is better to detect predators as wolves in images taking into account performance and speed due to it being a real-time vision module.

On the one hand, an SSD model was trained with pre-trained weights from VGG and Stochastic Gradient Descent (SGD) optimizer (as in [78]) with a learning rate of 0.0001. Training was completed during 100 epochs with a batch size of 16. The model achieves 92.90% of $mAP_{VOC}$ in the training set and 85.49% in the test set. Inference takes 80 ms on average, which corresponds with 12.5 FPS (in an NVIDIA GeForce RTX 2060).

On the other hand, multiple YOLO models were trained with an SGD optimizer configured with a learning rate of 0.01 (as in [80]) and a batch size of 4 during 50 epochs. Table 1 shows the obtained results of the different models. As it can be observed, YOLOv3 achieved the best results in $mAP_{COCO}$ with 88.63%, while the tiny model is the fastest one with 64 FPS also with an NVIDIA GeForce RTX 2060. With the newest version of YOLO, the extra-large model YOLOv5x yielded the highest $mAP_{COCO}$ with 88.24% but with the lower frame rate, whereas the nano, small and medium architectures are faster, achieving 64 FPS and keeping a mAP sligthly lower. Small architectures (nano YOLOv5n, small YOLOv5s and medium YOLOv5m) are lighter in weight, and therefore, the execution time is lower. Heavier architectures (large YOLOv5l and extra-large YOLOv5x) take longer to run but have higher precision in the results.

**Figure 6:**
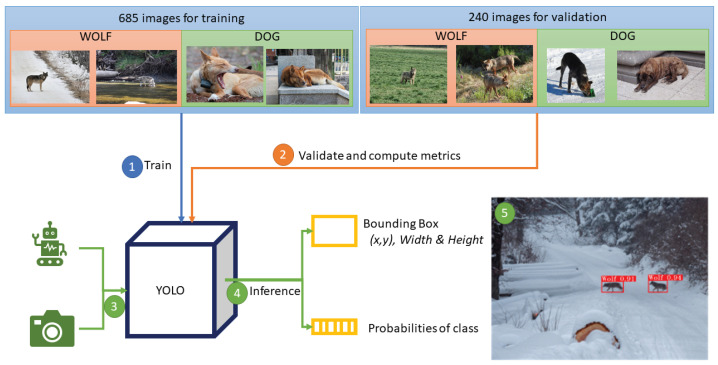
Complete scheme of the proposed vision module.

Figure 6 shows the scheme of the final module. Images are divided into training and validation using localisation: species from outside Europe are used for training and validation is performed with images of European species. First, the YOLO model is trained and then, metrics are calculated using the validation set (2nd step). Once the model is trained and evaluated as it is proposed in this paper, further steps involve integrating the model in an autonomous system such as a robot or in any device with a fixed camera, which acquires images (3rd step). The acquired images are processed by the model (4th step). Finally, identified threats such as the presence of wolves can be used to raise an alarm and inform the shepherd to avoid the area where they are located (5th step).

**Figure 7:**
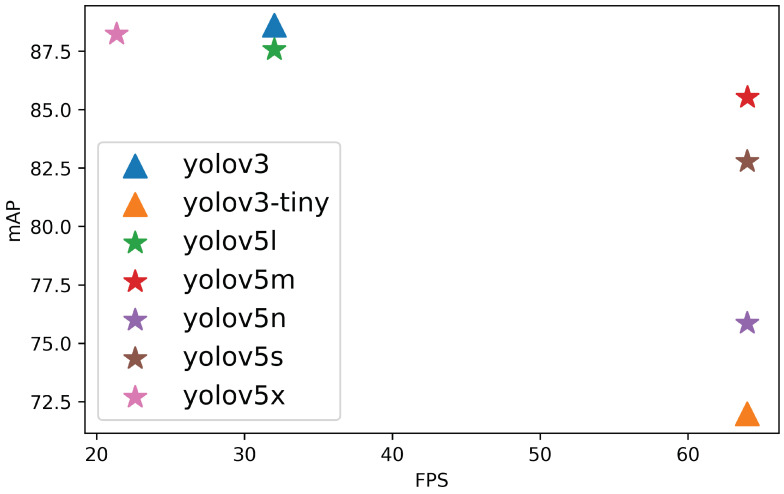
Results of the $mAP_{COCO}$ over the speed for YOLO models.

Further study of the results indicates that YOLO performs best in terms of accuracy and speed; that is, it is able to recognise wolves or wolf packs and distinguish them from dogs with high accuracy and in real time. Figure 7 shows a graph with the COCO metric and speed (FPS), where the best results are in the top right. We can determine that YOLOv5m (medium) is the best model in terms of speed and mAP, as it can process 64 FPS with 99.49% of $mAP_{VOC}$ and 85.53% of $mAP_{COCO}$. Observing these results, YOLOv5m is chosen to form the vision module.

**Figure 8:**
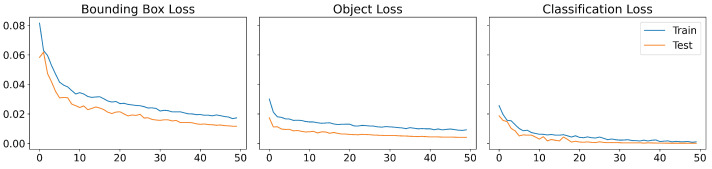
Loss functions of YOLOv5m during the training: bounding box loss with Mean Squared Error (**first graph**), object loss with Binary Cross-Entropy (**second graph**) and classification loss with Cross-Entropy (**third graph**).

**Figure 9:**
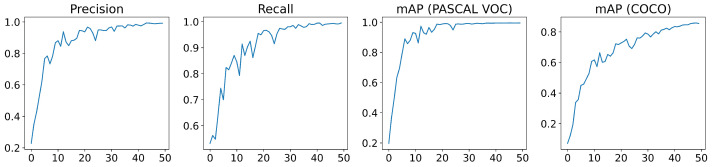
Metrics of YOLOv5m: precision (**first graph**), recall (**second graph**), $mAP_{VOC}$ which corresponds with PASCAL VOC metric (**third graph**) and $mAP_{COCO}$ which corresponds with COCO metric (**fourth graph**).

YOLOv5 employs a loss function composed by three losses: the bounding box loss, which uses a regression loss for object location (Mean Squared Error, MSE), the object loss, which obtains the confidence of object presence (Binary Cross-Entropy) and classification loss, which determines that the classification is correct (Cross-Entropy). Figure 8 displays the evolution of the loss functions during the training that shows the good behaviour of the model. Moreover, an evolution of the obtained metrics is shown in Figure 9, achieving a high performance after 30 epochs.

**Figure 10:**
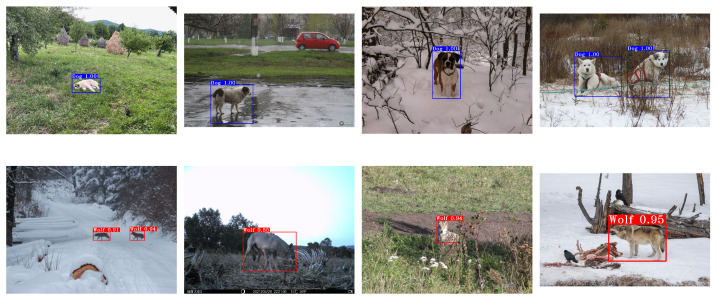
Samples of dogs (**upper row**) and wolves (**bottom row**) in Europe (**left**) and the rest of the world (**right**) with the detections yielded by YOLOv5m.

**Figure 11:**
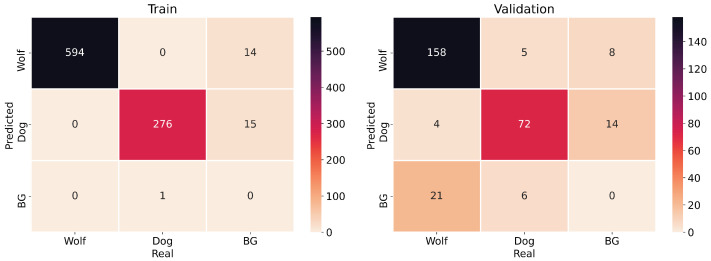
Confusion matrix for training (**left**) and validation (**right**) of YOLOv5m.

Finally, Figure 10 gathers some samples of the dataset with the objects detected by the YOLOv5m model. As it can be pointed out, there are images of the considered categories in which the objects to be detected, wolves and dogs, are at different distances to the camera and also deal with occlusions. Figure 11 shows the confusion matrix for training and validation data with an IoU greater than 0.5.

## 6. Discussion

There are approaches in the literature where researchers address similar problems of animal detection and classification. For instance, in [27], cattle are counted by analysing the images with a Mask R-CNN, obtaining 92% accuracy and AP for the detection of 91%, which is outperformed by the proposed method. In addition, using image processing and Mask R-CNN for counting animals, in [28], livestock, sheep and cattle are counted and classified, achieving a precision of 95.5% and $mAP_{40}$ values of 95.2%, 95% and 95.4% for livestock, sheep and cattle, respectively. Using thermal images, animals such as dogs, cats, deer, rhinos, horses and elephants are detected and classified with an $mAP_{VOC}$ of 75.98% with YOLOv3, 84.52% with YOLOv4 and 98.54% with Fast-RCNN [25]. In [24], the problem is turned into a classification as follows. First, a binary classification is performed to decide whether or not there are animals in the image, with an accuracy of 96.8%. If animals are detected, then a classification of the number of animals in the image is carried out, considering 1, 2, 3, 4, 5, 6, 7, 8, 9, 10, 11–50, or +51 individuals and achieving 63.1% top-1 accuracy.

According to the previously discussed methods, the proposed vision module outperforms the state-of-the-art results not only in precision and accuracy but also in speed to be able to couple to real-time systems. Table 2 summarises the comparison between the state-of-art methods explained above and the proposed method.

## 7. Conclusions

In this paper, a vision-based module to detect predators in pasture-based livestock farming and distinguish them from other species is proposed. This module can be deployed within on-site sheepdog robots and fixed cameras to assist shepherds in threat detection. First, we propose a system that can automatically generate datasets of different species through the iNaturalist API in order to obtain a module that can be used in any region depending on the existing predators. Focusing on a predator specie of the northwest of the Iberian Peninsula, namely the Iberian wolf, a particular dataset is obtained. The generated benchmark has the aim of providing data and an evaluation framework to test different algorithms to detect wolves, as a predator specie, and differentiate from other animals such as dogs, which have a similar physical appearance. These data can be automatically extended with the new predator and prey species of the region.

Then, multiple object detection models have been trained to establish which one achieves better results in a real-time module. According to the obtained results, the best results are achieved with YOLOv5m yielding an inference time of 15.62 ms, which allows 64 FPS. This model achieves a precision of 99.17% and a recall of 99.52% on the considered benchmark, outperforming other existing approaches, with an $mAP_{VOC}$ of 99.49% and $mAP_{COCO}$ of 85.53%. These results fulfil the requirements of a real-time detection module and improve state-of-the-art methods.

Future development lines involve integration into autonomous systems and data collection in the field. Information about potential threats will enable early warning alerts to be managed for herders in difficult-to-access terrain.

## Figures and Tables

**Table 1:** Results of the models over the test set, where $mAP_{VOC}$ is the PASCAL VOC metric and $mAP_{COCO}$ corresponds with the COCO metric. Inference time is measured in ms, and YOLOv3t is YOLOv3-tiny. Best results are highlighted in bold.

Model	Precision	Recall	${\mathrm{mAP}}_{\mathrm{VOC}}$	${\mathrm{mAP}}_{\mathrm{COCO}}$	Inference	FPS
SSD	85.49	93.33	85.49	47.87	80	12.5
YOLOv3	99.35	99.56	99.49	**88.63**	31.24	32.01
YOLOv3t	94.86	94.97	98.63	71.98	**15.62**	64.01
YOLOv5n	95.88	96.13	99.07	75.86	**15.62**	**64.02**
YOLOv5s	98.54	98.54	99.47	82.78	**15.62**	64.01
YOLOv5m	99.17	99.52	99.49	85.53	**15.62**	**64.02**
YOLOv5l	**99.47**	99.61	**99.50**	87.57	31.24	32.01
YOLOv5x	99.38	**99.73**	**99.50**	88.24	46.86	21.34

**Table 2:** Comparison of the state-of-the-art results with the proposed method for animal detection and count of different species.

Problem	Results
Holstein Friesian cattle detection and count [24]	Animal detection: accuracy of 96.8% Counting animals: 63.1% top-1 accuracy
Cattle count [27]	Counting animals: accuracy of 92% Bounding box prediction (localisation): AP of 91%
Livestock, Sheep, Cattle detection [28]	Precision rate: 95.5%, 96% and 95% Recall with IoU of 0.4: 95,2%, 95% and 95.4%
Animal detection in thermal images [25]	$mAP_{VOC}$ with YOLOv4: 75.98% $mAP_{VOC}$ with YOLOv3: 84.52% $mAP_{VOC}$ with Faster-RCNN: 98.54%
Wolf and Dog detection (Vision Module)	$mAP_{VOC}$ with YOLOv3: 99.49% (FPS: 32) $mAP_{VOC}$ with YOLOv5m: 99.49% (FPS: 64)

## Data Availability

All experiments and data are available in [71]. Images can be downloaded from iNaturalist API with the proposed system.

## Abbreviations

The following abbreviations are used in this manuscript:

CNN	Convolutional Neural Network
COCO	Common Objects in Context
FPS	Frame per second
IoU	Intersection over Union
LoRa	Long-Range Radio
mAP	mean Average Precision
PAZ	Perception for Autonomous Systems
PLF	Precision Livestock Farming
ROS	Robot Operating System
SGD	Stochastic Gradient Descent
SSD	Single-Shot MultiBox Detector
UGVs	Unmanned Ground Vehicles
VF	Virtual Fencing
VOC	Visual Object Classes
YOLO	You Only Look Once
